# Cognitive Impairment in Frail Hypertensive Elderly Patients: Role of Hyperglycemia

**DOI:** 10.3390/cells10082115

**Published:** 2021-08-17

**Authors:** Pasquale Mone, Jessica Gambardella, Antonella Pansini, Antonio de Donato, Giuseppe Martinelli, Eugenio Boccalone, Alessandro Matarese, Salvatore Frullone, Gaetano Santulli

**Affiliations:** 1 ASL Avellino, 83100 Avellino, Italy; antonellapansini87@gmail.com (A.P.); sfrullone@libero.it (S.F.); 2 Department of Mental and Physical Health and Preventive Medicine, University of Campania “*Luigi Vanvitelli*”, 80121 Naples, Italy; antoniodedonato88@gmail.com; 3 Department of Medicine (Cardiology), Wilf Family Cardiovascular Research Institute, Fleischer Institute for Diabetes and Metabolism (*FIDAM*), Albert Einstein College of Medicine, New York, NY 10461, USA; gambardellajessica@gmail.com; 4 International Translational Research and Medical Education Consortium (*ITME*), University “*Federico II*” of Naples, 80131 Naples, Italy; 5 ASL Naples, 80100 Naples, Italy; peppemartinelli85@gmail.com; 6 ASL Caserta, 81100 Caserta, Italy; eugeniob.85@gmail.com; 7 Cardarelli Hospital, 80131 Naples, Italy; alessandromatarese@yahoo.it; 8 Department of Molecular Pharmacology, Einstein Institute for Aging Research, Einstein-Sinai Diabetes Research Center (*ES*-*DRC*), Albert Einstein College of Medicine, New York, NY 10461, USA

**Keywords:** aging, antidiabetic drugs, metformin, endothelial cells, cognitive impairment, frailty, hypertension, hyperglycemia, anti-aging research, therapeutic strategies, age-related disease, metabolism

## Abstract

Endothelial dysfunction is a key hallmark of hypertension, which is a leading risk factor for cognitive decline in older adults with or without frailty. Similarly, hyperglycemia is known to impair endothelial function and is a predictor of severe cardiovascular outcomes, independent of the presence of diabetes. On these grounds, we designed a study to assess the effects of high-glucose and metformin on brain microvascular endothelial cells (ECs) and on cognitive impairment in frail hypertensive patients. We tested the effects of metformin on high-glucose-induced cell death, cell permeability, and generation of reactive oxygen species in vitro, in human brain microvascular ECs. To investigate the consequences of hyperglycemia and metformin in the clinical scenario, we recruited frail hypertensive patients and we evaluated their Montreal Cognitive Assessment (MoCA) scores, comparing them according to the glycemic status (normoglycemic vs. hyperglycemic) and the use of metformin. We enrolled 376 patients, of which 209 successfully completed the study. We observed a significant correlation between MoCA score and glycemia. We found that hyperglycemic patients treated with metformin had a significantly better MoCA score than hyperglycemic patients treated with insulin (18.32 ± 3.9 vs. 14.94 ± 3.8; *p* < 0.001). Our in vitro assays confirmed the beneficial effects of metformin on human brain microvascular ECs. To our knowledge, this is the first study correlating MoCA score and glycemia in frail and hypertensive older adults, showing that hyperglycemia aggravates cognitive impairment.

## 1. Introduction

Hypertension is one of the most common comorbidities, and is a recognized leading risk factor for cognitive decline [1,2,3,4,5,6,7,8]. Hypertension leads to chronic endothelial dysfunction, disrupting the integrity of endothelial cells (ECs) and contributing to oxidative stress, inflammation, and atherosclerosis [9,10,11,12,13,14,15,16].

Hyperglycemia (HG) is common in patients suffering from cardiovascular diseases [17], is an established independent predictor of severe outcomes, even in absence of frank diabetes mellitus (DM) [18,19], and has been proposed to exacerbate endothelial dysfunction in hypertensive subjects [20,21,22,23,24,25,26,27,28,29,30,31,32]. 

Frailty is a multidimensional condition due to reserve loss leading to both physical and cognitive impairment [33,34]. Frail older adults present a high-risk of adverse events including disability, hospitalization, and mortality [35]. Hence, it is very important to evaluate comorbidities and complications in order to reduce the incidence of cognitive and physical impairment; a thorough clinical assessment remains among the main strategies to obtain an early diagnosis (as well as a timely treatment) of cognitive impairment [36,37,38,39,40,41,42,43].

Metformin is an oral antidiabetic drug and its potential role in contrasting endothelial dysfunction and cognitive impairment currently represents a hot topic [44,45,46,47]; a recent trial on 80 subjects with mild cognitive impairment has shown significant improvements in verbal memory scores [48]; other investigators have underlined its beneficial effects, especially in older adults [47,49,50,51,52].

On these grounds, we aim to investigate the effects of HG and metformin on cognitive impairment in frail hypertensive patients and, in vitro, on human brain ECs.

## 2. Materials and Methods

### 2.1. Patients

We enrolled consecutive frail hypertensive elderly patients presenting to the ASL (local health providers managed by the Italian Ministry of Health) located in Avellino, Caserta, and Naples, Italy from April 2019 to April 2021. The following inclusion and exclusion criteria were applied:

*Inclusion criteria*:

1. A previous diagnosis of hypertension with no clinical or laboratory evidence of secondary causes;
2. Age > 65 years;
3. A frail status.

*Exclusion criteria*:

1. Age < 65 years;
2. Absence of frail status;
3. Absence of hypertension;
4. Left ventricular ejection fraction <25%, previous myocardial infarction, previous revascularization, or previous fibrinolytic therapy.

All patients underwent blood pressure measurement and blood analysis to assess glycemia. HG was defined by values ≥ 140 mg/dL, according to the American Diabetes Association [53]. Hypertension was defined as systolic blood pressure (SBP) ≥140 mm Hg, and/or diastolic blood pressure (DBP) ≥90 mm Hg on repeated measurements, or chronically treated hypertension with antihypertensive medications [54]. 

Based on glycemic values and medical treatment, we divided our population in the following four groups:

- Normoglycemic (NG) patients without DM (n: 51)
- NG patients with DM (n: 55)
- Metformin-treated HG patients (n: 53)
- Insulin-treated HG patients (n: 50)

### 2.2. Assessment of Cognitive Function

Cognitive function was assessed using the Montreal Cognitive Assessment (MoCA) test, which has been shown to be specific for the evaluation of cognitive domains (attention, concentration, memory, language, calculation, orientation and executive functions), and is generally considered one of the best tests to detect mild cognitive impairment [55,56,57].

### 2.3. Frailty Evaluation

A physical frailty assessment was performed following the Fried criteria [34,37]. A diagnosis of frailty status was made with at least three of the following five points:

- Weight loss (unintentional loss of ≥ 4.5 kg in the past year);
- Weakness (handgrip strength in the lowest 20% quintile at baseline, adjusted for sex and body mass index (BMI);
- Exhaustion (poor endurance and energy, self-reported);
- Slowness (walking speed under the lowest quintile adjusted for sex and height);
- Low physical activity level (lowest quintile of kilocalories of physical activity during the past week).

### 2.4. In Vitro Experiments

Human brain microvascular ECs were obtained from Neuromics (Minneapolis, MN, USA; catalog number: #HEC02). Cells were cultured in a standard humidified atmosphere (37 °C) containing 5% CO_2_, as we described [58]. In some experiments, cells were treated with metformin (0.1, 0.5, and 1 mM), adding glucose at 5 or 25 mM, or mannitol 20 mM as osmotic control, for 48 h. 

The endothelial permeability assay was performed as we described and validated [58,59], using fibronectin-coated transwell filters (Corning Inc., Corning, NY, USA). 

Levels of mitochondrial and cellular reactive oxygen species (ROS) were assessed using MitoSOX and CM-H_2_DCFDA dyes, respectively, as we previously described [60,61]. 

Cell death was evaluated using a Caspase-Glo^®^ 3/7 assay (Promega, Madison, WI, USA; catalog number: #G6321), as we described [62]. All reagents were from Millipore-Sigma (Burlington, MA, USA), unless otherwise stated. All experiments were conducted at least in triplicate by blinded investigators.

### 2.5. Statistical Analysis

Data are presented as mean ± SD. In an *a priori* power analysis, we calculated the number of patients required for the study to reject the null hypothesis 95% of the time (i.e., with a one-tailed type II error rate of 0.05) and with a two-tailed type I error at a 0.05 level of significance; sample size was calculated using the G-POWER software, yielding a minimum n of 50/group. 

We applied a dispersion model correlating MoCA score and glycemia; we performed a linear regression analysis with MoCA score as dependent variable to explore the impact of comorbidities. For the in vitro assays, we applied the two-way ANOVA followed by Tukey–Kramer multiple comparison test. 

Statistical significance was set at *p* < 0.05 and calculations were computed using SPSS 26 and GraphPad Prism 9.1.1.

## 3. Results

### 3.1. Population of Frail Hypertensive Patients

**Figure 1:**
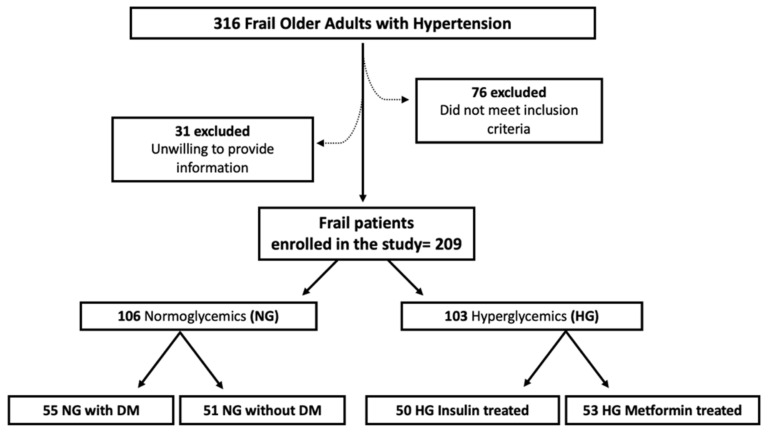
Study Flow Diagram. DM: diabetes mellitus; HG: hyperglycemic; NG: normoglycemic.

We evaluated a total of 316 frail patients with hypertension; 31 patients were unwilling to provide clinical information, and 76 subjects did not fulfill the inclusion and exclusion criteria (Figure 1). Hence, 209 patients entered the study: 106 NG and 103 HG subjects.

There were no significant differences in mean age, BMI, sex distribution, smoking habits, and comorbidities between the two groups (Table 1). All patients were taking antihypertensive drugs; the use of diuretics, angiotensin-converting enzyme inhibitors, angiotensin receptors blockers, beta-blockers, and calcium blockers was similar between the two groups (Table 1). Comorbidities are reported in Table 1, as well.

### 3.2. Glycemia Correlates with the MoCA Score in Frail Hypertensive Patients

**Figure 2:**
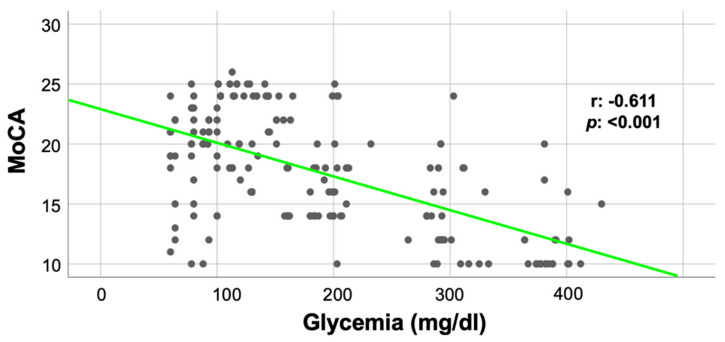
Correlation between MoCA score and glycemia.

Comparing NG and HG patients, we observed a significantly compromised cognitive function in the latter group in terms of MoCA score (NG: 19.95 ± 3.6 vs. HG: 15.78 ± 4.5; *p* < 0.05). Furthermore, as depicted in Figure 2, we found a strong correlation between the MoCA score and blood glucose levels (r: −0.611; *p*: <0.001).

To evaluate the impact of comorbidities on these findings, we performed a linear regression analysis using the MoCA score as dependent variable (Table 2). We observed a significant effect of age, diabetes, and cerebrovascular disease (CVD) (*p* < 0.001), alongside chronic kidney disease (*p*: 0.025).

### 3.3. The MoCA Score Is Significantly Higher in HG Patients Treated with Metformin Than in HG Patients Treated with Insulin

In order to evaluate the effect of metformin treatment on the MoCA score, we divided our patients according to their glycemic status and drug treatment, as follows: metformin-treated HG patients (MoCA score: 18.32 ± 3.9), insulin-treated HG patients (MoCA score: 14.94 ± 3.8), NG patients with DM (MoCA score: 17.45 ± 3.1), and NG patients without DM (MoCA score: 20.11 ± 3.3). 

**Figure 3:**
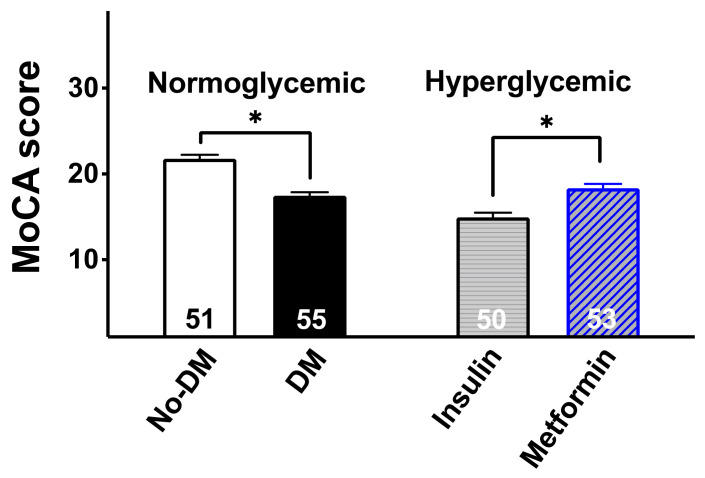
MoCA score in normoglycemic subjects without diabetes mellitus (No-DM), normoglycemic patients with diabetes mellitus (DM), hyperglycemic patients treated with insulin, and hyperglycemic patients treated with metformin. The number within each bar indicates the number of patients/group. Data are means ± SD; *: *p* < 0.05.

We found that the MoCA score in metformin-treated HG patients was significantly different from insulin-treated HG patients (*p* < 0.001, Figure 3).

### 3.4. In Vitro Assays

**Figure 4:**
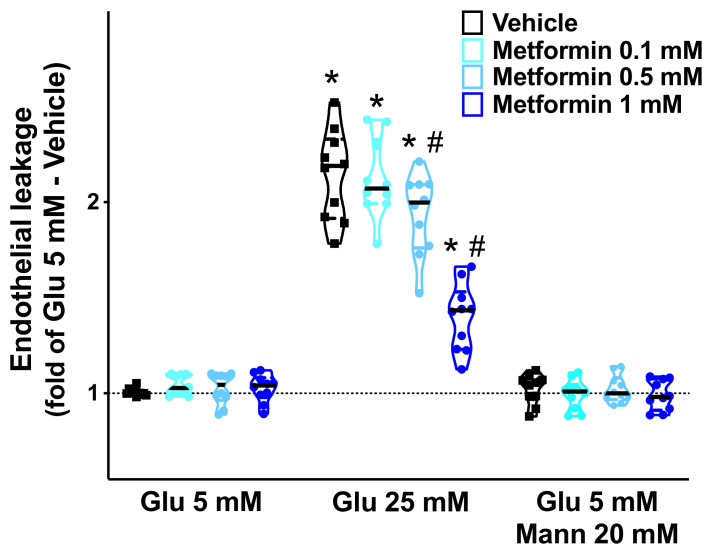
**Effects of metformin on endothelial permeability.** We assessed the dose-response relationship of metformin on the leakage of human brain microvascular endothelial cells, cultured at the indicated conditions (Glu: glucose; Mann: mannitol); *: *p* < 0.05 vs. Glu 5 mM; #: *p* < 0.05 vs. Vehicle.

**Figure 5:**
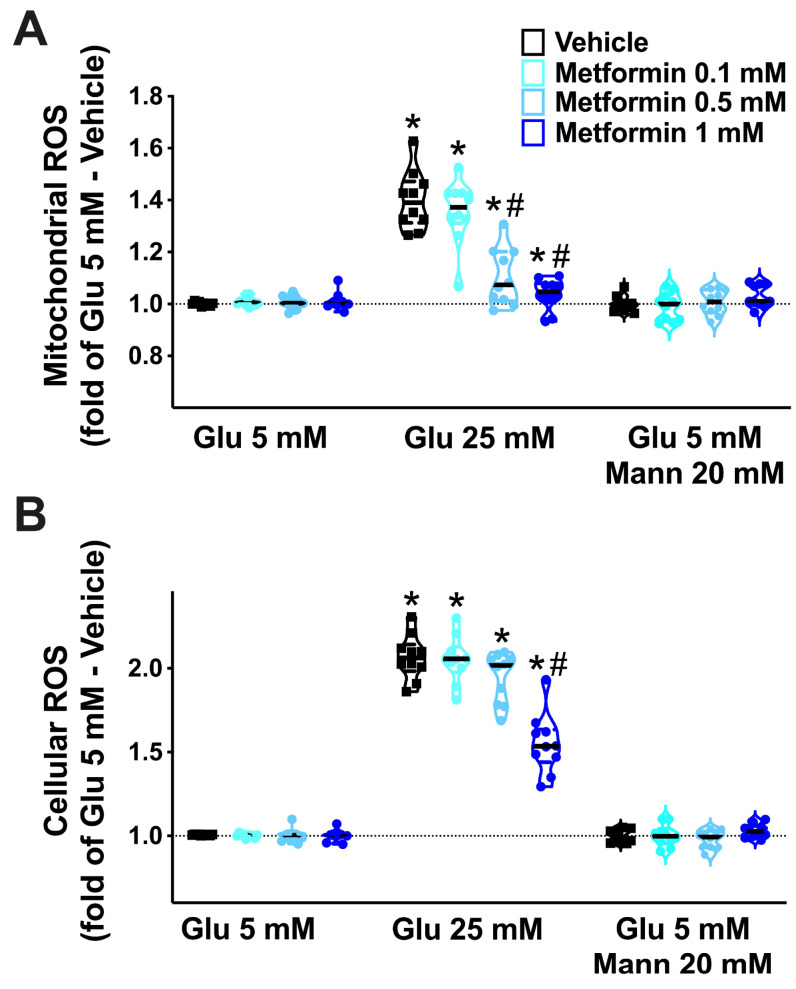
**Effects of metformin on the generation of reactive oxygen species (ROS).** We evaluated the effects of increasing doses of metformin on the production of mitochondrial (**A**) and cellular (**B**) ROS in human brain microvascular endothelial cells cultured at the indicated conditions (Glu: glucose; Mann: mannitol). *: *p* < 0.05 vs. Glu 5 mM; #: *p* < 0.05 vs. Vehicle.

**Figure 6:**
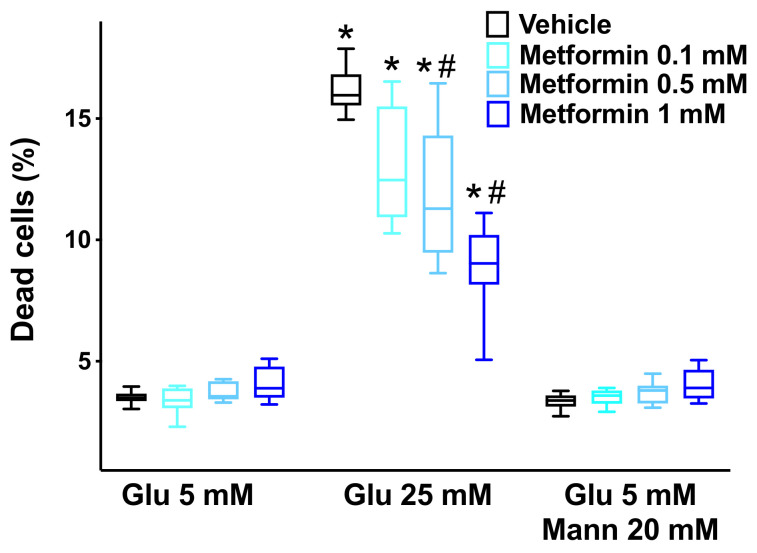
**Metformin attenuates high-glucose-induced cell death.** By using a luminogenic Caspase-Glo^®^ 3/7 assay, we evaluated the effects of increasing concentrations of metformin on the apoptosis of human brain microvascular endothelial cells induced by high-glucose (Glu: glucose; Mann: mannitol). The experiments were performed in triplicate. Box plots indicate upper/lower quartiles, the line in the middle of each box is the mean, and the whiskers represent the 5th–95th percentile range of values; *: *p* < 0.05 vs. Glu 5 mM; #: *p* < 0.05 vs. Vehicle.

High glucose concentrations have been shown to elicit a leak of the endothelial permeability barrier [63,64] and to increase ROS generation in ECs [23,65]. To test whether metformin could actually mitigate endothelial leakage and oxidative stress triggered by HG, we performed a series of in vitro dose-response experiments on human brain microvascular ECs. We found that metformin significantly attenuated the HG-induced endothelial leakage (Figure 4), reduced both mitochondrial (Figure 5A) and cellular (Figure 5B) ROS production, and prevented high-glucose-induced EC apoptosis (Figure 6).

## 4. Discussion

Our results indicate that HG subjects have a lower MoCA score than the NG ones. These data must be contextualized in a frailty condition with hypertension, in which cognitive impairment has a strong impact on functional disability and loss of independence [66,67]. The management of frailty in older adults is extremely challenging [68,69,70] and comorbidities such as hypertension play an instrumental role in increasing the risk of mortality, hospitalization, and disability [71,72]. In this scenario, such a delicate balance is heavily disturbed by HG.

Herein, we analyzed the relationship between the MoCA score and glycemia observing a strong correlation (r: −0.611; *p* < 0.001). To further confirm these results, we performed a linear regression analysis to verify the impact of comorbidities, using the MoCA score as a dependent variable; we found a significant association for age, diabetes, and CVD. Taken together, these data strongly suggest that the achievement of an optimal glycemic control could reduce cognitive impairment in frail and hypertensive older adults.

In this scenario, metformin-treated HG patients presented a better cognitive function than insulin-treated HG patients. These results are consistent with previous reports confirming the beneficial effects of metformin in cognitive impairment [47,73,74,75]. Other studies have instead shown no significant action [76] or even a detrimental effect [77] of metformin on cognition, although the mechanisms underlying such findings were not explored. These contrasting results could be attributed to several factors, including the disparities in the studied populations. Of note, in our population there were no significant differences between metformin-treated HG patients and NG patients with diabetes, and this aspect strengthens the pivotal role of metformin in counteracting cognitive impairment in our population. Our in vitro results showing a dose-dependent beneficial action of metformin on high-glucose- induced endothelial leakage, oxidative stress, and cell death, provide a molecular mechanism underlying our clinical observations. Of note, these findings are consistent with previous reports [78,79,80,81,82,83,84,85,86,87].

Hypertension determines alterations of the endothelium causing oxidative stress, increased vascular permeability, and atherosclerosis [88,89,90,91]. We hypothesized that metformin treatment, among its numerous pleiotropic effects [92,93], could attenuate endothelial dysfunction caused by HG, a crucial feature in hypertensive older adults with frailty [94,95,96]. Furthermore, metformin may modulate and reduce the pathologic mechanisms triggered by insulin resistance [79,97,98]. Indeed, metformin has been proposed to be an anti-aging drug and has been associated with a lower risk of frailty in community-dwelling veterans [99,100].

## 5. Conclusions

To the best of our knowledge, this study is the first to correlate MoCA score and glycemia in frail and hypertensive older adults, showing that HG significantly exacerbates cognitive impairment in this class of patients. Although we found beneficial effects of metformin treatment, future dedicated studies with larger samples and longer follow-up are required to confirm our results.

## Figures and Tables

**Table 1:** Clinical characteristics of our population.

	NG Patients	HG Patients
N	106	103
Mean age (years)	75.0 ± 6.5	76.0 ± 6.1
BMI (kg/m^2^)	27.1 ± 1.9	27.3 ± 1.8
SBP (mmHg)	128.9 ± 12.4	129.2 ± 11.8
DBP (mmHg)	78.8 ± 6.6	79.2 ± 6.3
Heart rate (bpm)	87.5 ± 8.3	88.2 ± 8.2
***Comorbidities***		
Diabetes, n (%)	56 (52.0)	103 (100)
Dyslipidemia, n (%)	66 (63.0)	66 (64.0)
CVD, n (%)	29 (30.0)	31 (30.5)
COPD, n (%)	38 (36.5)	39 (38.0)
CKD n (%)	43 (41.0)	44 (43.0)
Current smokers, n (%)	44 (41.5)	41 (40.0)
***Active treatments***		
β-blockers, n (%)	61 (58.0)	57 (56.0)
ACE inhibitors, n (%)	73 (69.0)	70 (68.0)
Angiotensin receptor blockers, n (%)	35 (33.0)	33 (32.0)
Calcium inhibitors, n (%)	24 (23.0)	28 (27.0)
Statins, n (%)	57 (54.0)	54 (53.0)
Diuretics, n (%)	8 (8.0)	11 (11.0)
Aspirin, n (%)	46 (44.0)	50 (49.0)
***Laboratory analyses***		
Plasma glucose (mg/dL)	124.6 ± 11.8	207.3 ± 18.6
Total cholesterol (mg/dL)	193.9 ± 19.8	192.6 ± 20.2
Creatinine (mg/dL)	1.0 ± 0.1	1.0 ± 0.1

ACE: angiotensin converting enzyme; BMI: body mass index; COPD: chronic obstructive pulmonary disease; CKD: chronic kidney disease; CVD: cardiovascular disease; DBP: diastolic blood pressure; HG: hyperglycemic; NG: normoglycemic; SBP: systolic blood pressure.

**Table 2:** Linear regression analysis with the MoCA score as dependent variable.

	B	Standard Error	Beta	t	*p*
**Age**	−0.247	0.042	−0.340	−5.893	<0.001
**Diabetes**	−4.306	0.544	−0.427	−7.923	<0.001
**CVD**	−4.349	0.505	−0.469	−8.615	<0.001
**Hyperlipidemia**	−0.619	0.483	−0.065	−1.281	0.202
**CKD**	−1.213	0.537	−0.128	−2.257	0.025
**COPD**	1.861	0.522	0.197	3.566	0.050

COPD: chronic obstructive pulmonary disease; CKD: chronic kidney disease; CVD: cardiovascular disease.

## Data Availability

All data are contained within the article.
